# The Association Between Serum Ferritin Concentration and Visceral Adiposity Estimated by Whole-Body DXA Scan in Women With Polycystic Ovary Syndrome

**DOI:** 10.3389/fendo.2019.00873

**Published:** 2020-01-08

**Authors:** Agnieszka Adamska, Agnieszka Łebkowska, Anna Krentowska, Marcin Adamski, Irina Kowalska

**Affiliations:** ^1^Department of Endocrinology, Diabetology and Internal Medicine, Medical University of Białystok, Białystok, Poland; ^2^Department of Internal Medicine and Metabolic Diseases, Medical University of Białystok, Białystok, Poland; ^3^Faculty of Computer Science, Bialystok University of Technology, Białystok, Poland

**Keywords:** ferritin, DXA, PCOS, VAT, insulin resistance

## Abstract

**Objective:** Women with polycystic ovary syndrome (PCOS) are characterized by insulin resistance and higher prevalence of obesity. Serum ferritin is increased in obesity and is associated with insulin resistance. The aim of the present study was to evaluate the relationships between serum ferritin concentration with insulin resistance and body composition estimated by dual-energy X-ray absorptiometry (DXA) in PCOS women in comparison to the control group.

**Patients and Methods:** One hundred four women were enrolled to the study−65 women with PCOS and 39 women matched for age and BMI as a control group. Serum ferritin concentration and oral glucose tolerance test (OGTT) were performed. Homeostasis model assessment of insulin resistance (HOMA-IR) was calculated. DXA was performed to estimate fat, fat-free mass, and visceral adipose tissue (VAT).

**Results:** Women with PCOS have higher serum concentration of ferritin (*p* = 0.002), insulin at baseline (*p* = 0.03), at 60 min of OGTT (*p* = 0.01), at 120 min of OGTT (*p* = 0.004), HOMA-IR (*p* = 0.03), and VAT (*p* = 0.0001) in comparison to the control group. We observed a relationship of serum ferritin with insulin concentration at baseline (*r* = 0.25, *p* = 0.04) and at 120 min of OGTT (*r* = 0.31, *p* = 0.01) and with HOMA-IR (*r* = 0.30, *p* = 0.01) in the PCOS group. We noticed an association between serum ferritin concentration and VAT (*r* = 0.42, *p* = 0.001), trunk fat mass (*r* = 0.25, *p* = 0.04), and android fat mass (*r* = 0.25, *p* = 0.04) in the PCOS group. Multiple regression analysis revealed that ferritin (*p* = 0.02, β = 0.17), insulin at baseline (*p* = 0.001, β = 0.30), glucose at the 120 min of OGTT (*p* = 0.007, β = 0.26), and triglycerides (*p* = 0.001, β = 0.33) were independent predictors of VAT amount in PCOS women.

**Conclusions:** Elevated serum ferritin concentration is connected with insulin resistance as well as with DXA-estimated VAT, android, and trunk fat mass in PCOS women, and could be a marker of metabolic dysfunction.

## Introduction

Polycystic ovary syndrome (PCOS) is a common disorder characterized by hyperandrogenism (biochemical and/or clinical), ovulatory dysfunction, and characteristic changes in the ovaries (1). Moreover, approximately 50% of PCOS women are overweight or obese (2). Insulin resistance (IR) and hyperinsulinemia are connected with weight gain and development of obesity in this population (3). Women with PCOS also have altered fat distribution and greater tendency to increased visceral adipose tissue (VAT) accumulation compared to BMI-matched general population (4), even in the normal range for BMI (5). A number of studies have demonstrated that increased amount of VAT, which is metabolically more active than subcutaneous adipose tissue (SAT), is connected with dyslipidemia, hypertension, insulin resistance, and type 2 diabetes. Accordingly, it has been reported that elevated level of androgens is connected with abdominal fat deposition (6).

The gold standard for the assessment of fat distribution (VAT and SAT) is magnetic resonance; however, this technique is expensive and time consuming. Other methods are also used, e.g., ultrasonography, useful in general population, or dual-energy X-ray absorptiometry (DXA) (7). It has been shown that DXA may be helpful in the early detection of visceral obesity. Moreover, DXA showed high reproducibility, making this method suitable for repeated measurements in the same individual over time (8).

Serum ferritin is essential for iron homeostasis and is a non-invasive indicator of total body iron status (9). Serum ferritin level increases in the state of chronic kidney disease (10), autoimmune disorders (11), acute and chronic inflammation, cardiovascular disease (12), and metabolic syndrome (13). Moreover, elevated serum ferritin levels have been observed in PCOS women (14–16). In a recent meta-analysis, it has been shown that serum ferritin concentration is connected with fasting plasma glucose, serum insulin concentration (17), and plasma triglycerides (TG) level (18). Additionally, studies revealed that serum ferritin levels are connected with homeostasis model assessment of insulin resistance (HOMA-IR) in a non-diabetic population but not in patients with impaired glucose tolerance (19–22). Therefore, serum ferritin has been proposed as an early marker of insulin resistance (21). Accordingly, it has been shown that serum ferritin concentration was significantly associated with fat distribution in subjects with and without diabetes (23). Moreover, different markers of iron metabolism in adipose tissue are associated with insulin action (24). Therefore, body fat distribution may be significantly associated with body iron stores as reflected by serum ferritin concentrations.

In the face of the fact that women with PCOS are characterized by insulin resistance, abdominal fat accumulation and elevated serum level of ferritin, we aimed to test the relationships between these components. Therefore, the purpose of the present study was to investigate the relationships between serum ferritin concentration with insulin resistance and body composition estimated by DXA in PCOS women in comparison to the control group.

## Subjects and Methods

### Ethics Approval

All procedures performed in studies involving human participants were in accordance with the ethical standards of the institutional and national research committee (Ethics Committee of Medical University of Białystok, Białystok, Poland) and with the 1964 Helsinki declaration and its later amendments or comparable ethical standards. Informed consents were obtained from all patients after full explanation of the purpose and nature of all procedures used. All the enrolled patients participated in the research voluntarily and freely. All the procedures were performed in accordance with the relevant guidelines and regulations.

### Subjects

One hundred four women were enrolled to the study−65 women with PCOS and 39 women matched for age and BMI as a control group. Women with PCOS were recruited from the Department of Endocrinology, Diabetology and Internal Medicine, Medical University of Białystok, as well as from the Department of Internal Medicine and Metabolic Diseases, Medical University of Białystok and among students between January 2016 year to July 2018 year. Students were enrolled as control subjects if they did not meet exclusion criteria. PCOS was diagnosed according to the 2003 Rotterdam ESHRE/ASRM PCOS Consensus Workshop Group diagnostic criteria (25). PCOS was recognized when at least two of the following three criteria were met: (1) clinical and/or biochemical hyperandrogenism, (2) oligomenorrhea or anovulation, (3) polycystic ovaries on ultrasound (>12 follicles measuring 2–9 mm in diameter or ovarian volume >10 ml in at least one ovary). Exclusion criteria included the following: thyroid disorders, morbid obesity, hyperlipidemia, cardiovascular disease, liver disease, autoimmune disease; other causes of irregular menstrual cycles and/or androgen excess (i.e., hyperprolactinemia, Cushing's syndrome, late-onset congenital adrenal hyperplasia, other diseases of the adrenal glands, pregnancy and breastfeeding); type 1 or type 2 diabetes; chronic or acute infection (within the previous 30 days), women with elevated high-sensitivity C-reactive protein (hsCRP) were excluded; any other serious medical problem, hormonal contraception, and/or anti-androgen therapy (within the previous 6 months). Moreover, participants taking any medications (e.g., drugs affecting lipid and glucose metabolism) were excluded from the study. All women were non-smoking. Blood samples were collected after an overnight fast 3–5 days after a spontaneous menses or independent of cycle phase in the presence of amenorrhea in the PCOS women. In the control group, the studies were performed during the early follicular phase (3–5 days) of their menstrual cycles.

### Study Protocol

All study participants—PCOS women and controls—followed the same study protocol. Clinical examination was performed in all women. Clinical hyperandrogenism was evaluated using the modified Ferriman–Gallwey score for hirsutism (more than eight points was considered as clinical hyperandrogenism) and/or presence of acne. Oligo/amenorrhea and anovulation were considered when women had fewer than six menses during the previous year. Ultrasound scans were done for all the patients by the same gynecologist with a 5–9 MHz transvaginal transducer (Voluson 730 Expert GE Healthcare) in the early follicular phase of the menstrual cycle. Ovarian volume was calculated using the simplified formula for a prolate ellipsoid (26). Studies were performed in the PCOS group 3–5 days after a spontaneous menses or independent of cycle phase in the presence of amenorrhea. In the control group, the studies were performed during the early follicular phase (3–5 days) of their menstrual cycles.

### Oral Glucose Tolerance Test and Assays of Metabolic Biomarkers

Oral glucose tolerance test (OGTT) was carried out using 75 g of glucose after an 8- to 12-h overnight fast. All subjects were advised to follow their normal diet for at least 3 days prior to the test.

### Anthropometric Measurements

BMI was calculated as body weight in kilograms divided by height in meters squared (kg/m^2^). Waist circumference was measured at the smallest circumference between the rib cage and the iliac crest, with the subject in the standing position. The hip circumference measurement was obtained at the maximum perimeter at the level of the femoral trochanters. Systolic and diastolic blood pressure was recorded.

### Biochemical Analyses

Plasma glucose level was measured immediately by the enzymatic reference method with hexokinase (Cobas c111, Roche Diagnostic Ltd., Switzerland). Serum insulin concentration was assayed by immunoradiometric method (DIAsource ImmunoAssays S.A., Belgium). The minimum detectable concentration was 1 μIU/ml, and the intra-assay and inter-assay coefficients of variation (CVs) were below 2.2 and 6.5%, respectively. In this method, human and animal proinsulins present no cross-reactions. Plasma total cholesterol (TC), HDL-cholesterol, and TG were assessed by an enzymatic, colorymetric method (Cobas c111, Roche Diagnostic Ltd., Switzerland). Plasma LDL-cholesterol was calculated according to Friedewald's formula. Serum follicle-stimulating hormone (FSH), luteinizing hormone (LH), and prolactin (PRL) concentrations were measured by immunoradiometric method (DIAsource ImmunoAssays S.A., Belgium). The intra-assay and inter-assay coefficients of variation were below 3.9 and 8% for LH, below 2 and 4.4% for FSH, and below 5.2 and 9.2% for PRL, respectively. Total testosterone concentration was determined by radioimmunoassay (DIAsource ImmunoAssays S.A., Belgium); the minimum detectable concentration was 0.05 ng/ml, and the intra-assay and inter-assay coefficients of variation were estimated at 3.3 and 4.8%, respectively. Serum sex hormone–binding globulin (SHBG) was measured by immunoradiometric assay (ZenTech, Angleur, Belgium). The intra-assay and inter-assay coefficients of variation for SHBG were below 5.2 and 5.8%, respectively. Free androgen index (FAI) was calculated as serum total testosterone (nmol/L) × 100/SHBG (nmol/L) ratio (27). Serum thyroid-stimulating hormone (TSH) concentration was estimated by immunoradiometric method. The sensitivity and CVs for TSH assays were identical, as reported previously (28). Serum concentration of hsCRP serum was measured by highly sensitive immuno-turbidimetric assay (Cobas c111, Roche Diagnostic Ltd., Switzerland). Serum ferritin concentration was estimated with chemiluminescence method (Cobas e411, Roche Diagnostic Ltd., Switzerland). The minimum detectable concentration was 0.5 ng/ml and the intra-assay and inter-assay coefficients of variation (CVs) were below 9.1 and 11.2%, respectively.

### Calculations

The homeostasis model assessment of insulin resistance (HOMA-IR) was calculated according to the formula: (fasting insulin (μIU/ml) × fasting plasma glucose (mmol/L))/22.5 (29).

Whole-body insulin sensitivity was calculated by Matsuda index according to the formula: 10.000/square root of [fasting glucose (mmol/l) × fasting insulin (μIU/ml)] × (mean glucose (mmol/l) × mean insulin (μIU/ml) during OGTT) (30).

### Body Composition Analyses

Body composition analyses were performed using DXA (GE Healthcare Lunar) at the Clinical Research Centre, Medical University of Bialystok, by qualified physicians. Calibration was performed before every patient. Women were positioned in supine position, feet strapped together, and hands placed flat on the table adjacent to the side of the body. Average scanning time was approximately 8 min. With this method, body composition consisting of body fat (kg) and lean (kg) soft tissue was estimated. For each region of the whole body (head, trunk, arms, and legs), fat and lean body mass were determined. Software CoreScan estimated VAT within the android region, expressed as mass (g). DXA assessed lean mass and fat mass with the precision (coefficient of variation) of 2.0 and 8.0%, respectively.

### Statistical Analysis

Statistical analyses were performed using the STATISTICA 10.0 software. Before analyses were carried out, the distribution of the variables was tested for normality using Shapiro-Wilk *W* test and non-normally distributed parameters were logarithmically transformed. The following variables were transformed using logarithm function: TG, HOMA-IR, and data derived from DXA. For the purpose of the data presentation, absolute values are shown in the *Results* section. The differences between clinical and biochemical parameters between the PCOS group and control women were evaluated with an unpaired Student's *t* test. Variables were presented as mean ± standard deviation (SD). The relationships between the variables were evaluated using Pearson correlation coefficient. Afterwards, multivariate regression analysis was performed to identify independent relationships. The level of significance was accepted at *p* < 0.05.

## Results

### Basic Characteristic of the Studied Groups

Table 1 presents the main clinical and biochemical characteristics of the studied subjects. The PCOS group and the control group were matched for age, BMI, and waist and hip circumference (all *p* > 0.05). The groups did not differ in terms of plasma glucose during OGTT, lipids, and serum TSH concentrations (all *p* > 0.05) (Table 1). DXA analysis revealed no differences in total fat mass, arms fat mass, android and gynoid fat mass, total lean mass and arms lean mass, total fat-free mass, arms and legs fat-free mass, and gynoid and android fat-free mass (all *p* > 0.05). However, the PCOS group was characterized by higher amount of VAT (*p* = 0.0001) (Table 2).

**Table 1:** Clinical and biochemical characteristics of the studied groups.

	**Control group (*n* = 39)**	**PCOS (*n* = 65)**	***p***
Age (years)	26.2 ± 4.5	25.1 ± 4.4	0.23
BMI (kg/m^2^)	23.7 ± 3.1	25.2 ± 1.1	0.08
Waist circumference (cm)	81.6 ± 9.0	84.6 ± 12.5	0.19
Hip circumference (cm)	100.7 ± 7.8	101.6 ± 9.6	0.60
Follicle-stimulating hormone (IU/L)	5.5 ± 1.4	5.2 ± 1.4	0.44
Luteinizing hormone (IU/L)	4.5 ± 1.4	4.8 ± 1.9	0.47
Total testosterone (ng/ml)	0.58 ± 0.2	0.67 ± 0.2^*^	0.02
SHBG (nmol/L)	63.7 ± 28.0	49.6 ± 37.9^*^	0.04
FAI	3.9 ± 2.9	6.4 ± 4.8^*^	0.003
Prolactin (ng/ml)	16.1 ± 18.2	14.9 ± 11.8	0.70
Glucose 0′ OGTT (mg/dl)	91 ± 7.6	92 ± 7.5	0.59
Glucose 30′ OGTT (mg/dl)	130 ± 23.3	138 ± 25.7	0.09
Glucose 60′ OGTT (mg/dl)	110 ± 26.8	115 ± 36.8	0.05
Glucose 120′ OGTT (mg/dl)	92 ± 21.6	97 ± 23.5	0.22
Insulin 0′ OGTT (μIU/ml)	9.4 ± 5.7	11.4 ± 5.1^*^	0.03
Insulin 30′ OGTT (μIU/ml)	69.8 ± 52.1	80.2 ± 45.2	0.27
Insulin 60′ OGTT (μIU/ml)	51.5 ± 43.8	75.8 ± 50.1^*^	0.01
Insulin 120′ OGTT (μIU/ml)	28.3 ± 15.5	54.4 ± 57.2^*^	0.006
HOMA-IR	2.1 ± 1.2	2.7 ± 1.4	0.03
Matsuda index	6.2 ± 1.7	5.2 ± 2.5^*^	0.04
Total cholesterol (mg/dl)	169.8 ± 24.3	173.8 ± 25.8	0.37
HDL-cholesterol (mg/dl)	66.8 ± 21.5	65.1 ± 17.4	0.65
LDL-cholesterol (mg/dl)	89.1 ± 23.1	93.5 ± 21.7	0.32
TG (mg/dl)	66.6 ± 29.3	75.5 ± 45.4	0.27
TSH (uIU/ml)	1.9 ± 0.73	2.1 ± 0.96	0.50
Ferritin (ng/ml)	31.1 ± 22.4	47.3 ± 30.6^*^	0.004

*Data are presented as mean ± SD. Differences between the groups are derived from Student's t-test*.

* *p < 0.05 in PCOS women vs. control group*.

*BMI, body mass index; TG, triglycerides; OGTT, oral glucose tolerance test; FSH, follicle-stimulating hormone; LH, luteinizing hormone; FAI, free androgen index; SHBG, sex hormone binding globulin; HOMA-IR; homeostasis model assessment of insulin resistance, TSH, thyroid-stimulating hormone*.

**Table 2:** Body composition parameters estimated with DXA of the studied groups.

	**Control group (*n* = 39)**	**PCOS (*n* = 65)**	***p***
Arms fat mass (kg)	2.4 ± 0.6	2.7 ± 1.0	0.10
Android fat mass (kg)	1.5 ± 0.7	1.9 ± 1.1	0.07
Gynoid fat mass (kg)	4.4 ± 1.1	4.5 ± 1.5	0.61
Total fat mass (kg)	22.0 ± 0.6	24.4 ± 0.9	0.17
Arms lean mass (kg)	4.1 ± 0.6	4.3 ± 0.6	0.14
Gynoid lean mass (kg)	6.5 ± 0.8	6.6 ± 0.7	0.50
Total lean mass (kg)	42.4 ± 4.6	42.1 ± 4.7	0.78
Arms fat-free mass (kg)	4.5 ± 0.6	4.4 ± 0.7	0.17
Legs fat-free mass (kg)	16.1 ± 2.1	16.0 ± 2.4	0.80
Gynoid fat-free mass (kg)	6.8 ± 0.8	6.9 ± 0.8	0.55
Android fat-free mass (kg)	2.9 ± 0.4	2.9 ± 0.3	0.80
Total fat-free mass (kg)	44.6 ± 4.7	44.8 ± 4.9	0.84
VAT mass (g)	249.4 ± 259.7	427.9 ± 476.1^*^	0.03

*Data are presented as mean ± SD. Differences between the groups are derived from Student's t-test*.

* *p < 0.05 in PCOS women vs. control group*.

*DXA; dual energy x-ray absorptiometry, VAT; visceral adipose tissue*.

Women with PCOS presented higher serum concentrations of total testosterone (*p* = 0.002), FAI (*p* = 0.001), and lower serum concentration of SHBG (*p* = 0.002) than the control group (Table 1).

Serum insulin concentration at baseline and at 60 min of OGTT and 120 min of OGTT (*p* = 0.03, *p* = 0.01, *p* = 0.004; respectively), as well as HOMA-IR (*p* = 0.03) were higher in the PCOS group in comparison to women without PCOS, whereas the Matsuda index was lower in PCOS women vs. the control group (*p* = 0.04) (Table 1).

### Relationship Between Serum Ferritin Concentrations With Estimated Parameters

Higher serum concentrations of ferritin were observed in PCOS women as compared to the control group (*p* = 0.002) (Table 1).

We observed a positive relationship between serum ferritin concentration and plasma TG concentration (*r* = 0.25, *p* = 0.04), FAI (*r* = 0.45, *p* = 0.0001), serum insulin concentration at baseline (*r* = 0.25, *p* = 0.04), and 120 min of OGTT (*r* = 0.31, *p* = 0.01), as well as with HOMA-IR (*r* = 0.30, *p* = 0.01), only in the PCOS group.

No statistically significant relationship was observed between serum ferritin concentration and BMI, waist and hip circumference in the whole group, as well as in PCOS and control group analyzed separately (all *p* > 0.05). Additionally, we did not observe relationships between serum ferritin concentration and total fat mass, arms fat mass, gynoid fat mass, total lean mass and arms lean mass, total fat-free mass, arms and legs fat-free mass, and gynoid and android fat-free mass measured by DXA in PCOS women, as well as in the control group (all *p* > 0.05) (Table 3).

**Table 3:** Relationship between serum ferritin concentration and body composition parameters estimated with DXA of the studied groups.

	**Serum ferritin concentration**	
	**Control group (*n* = 39)**	**PCOS (*n* = 65)**
Arms fat mass (kg)	*r* = 0.24, *p* = 0.13	*r* = 0.10, *p* = 0.4
Trunk fat mass (kg)	*r* = 0.27, *p* = 0.08	*r* = 0.25, *p* = 0.04^*^
Android fat mass (kg)	*r* = 0.32, *p* = 0.04^*^	*r* = 0.25, *p* = 0.04^*^
Gynoid fat mass (kg)	*r* = 0.14, *p* = 0.36	*r* = 0.06, *p* = 0.58
Total fat mass (kg)	*r* = 0.24, *p* = 0.14	*r* = 0.11, *p* = 0.34
Total lean mass (kg)	*r* = −0.02, *p* = 0.98	*r* = −0.11, *p* = 0.37
Arms fat-free mass (kg)	*r* = 0.03, *p* = 0.81	*r* = −0.09, *p* = 0.45
Gynoid fat-free mass (kg)	*r* = 0.03, *p* = 0.81	*r* = −0.07, *p* = 0.55
Android fat-free mass (kg)	*r* = 0.04, *p* = 0.97	*r* = −0.04, *p* = 0.55
Total fat-free mass (kg)	*r* = 0.06, *p* = 0.96	*r* = −0.11, *p* = 0.36
VAT mass (g)	*r* = 0.38, *p* = 0.01^*^	*r* = 0.42, *p* = 0.01^*^

Data are derived from Pearson correlation coefficient. The level of significance was accepted at

* *p < 0.05*. *DXA; dual energy x-ray absorptiometry, VAT; visceral adipose tissue*.

In both PCOS group and control group, there was a significant relationship between serum ferritin concentration and VAT (*r* = 0.42, *p* = 0.001 and *r* = 0.38, *p* = 0.01, respectively). Moreover, serum ferritin concentration was positively associated with trunk fat mass (*r* = 0.25, *p* = 0.04) and android fat mass (*r* = 0.25, *p* = 0.04) in patients with PCOS. In the control group, serum ferritin concentration correlated with android fat mass (*r* = 0.32, *p* = 0.04) (Table 3).

### Relationship Between VAT and Estimated Parameters

In linear regression analysis, VAT was related to plasma TG concentration (*r* = 0.74, *p* < 0.01), plasma LDL-cholesterol concentration (*r* = 0.25, *p* = 0.04), plasma HDL-cholesterol concentration (*r* = −0.57, *p* < 0.01), plasma glucose concentration at baseline (*r* = 0.31, *p* = 0.01), and at the 120 min of OGTT (*r* = 0.62, *p* < 0.01), serum insulin concentration at baseline (*r* = 0.66, *p* < 0.01), and at the 120 min of OGTT (*r* = 0.59, *p* < 0.01) and with HOMA-IR (*r* = 0.68, *p* < 0.01) and Matsuda index (*r* = −0.57, *p* < 0.01) in PCOS women. We found a relationship between VAT and serum concentration of insulin at baseline (*r* = 0.75, *p* < 0.01), and at the 120 min of OGTT (*r* = 0.48, *p* = 0.02) and with HOMA-IR (*r* = 0.77, *p* < 0.01) and Matsuda index (*r* = −0.60, *p* < 0.01) in the control group (Table 4).

**Table 4:** Relationship between VAT and biochemical parameters in PCOS and control group.

	**VAT**	
	**Control group (*n* = 39)**	**PCOS (*n* = 65)**
Total cholesterol	*r* = 0.16, *p* = 0.32	*r* = 0.08, *p* = 0.48
TG	*r* = 0.15, *p* = 0.35	*r* = 0.74, *p* < 0.01^*^
HDL-cholesterol	*r* = 0.16, *p* = 0.30	*r* = −0.57, *p* < 0.01^*^
LDL-cholesterol	*r* = −0.24, *p* = 0.88	*r* = 0.25, *p* = 0.04^*^
Glucose 0′ OGTT	*r* = 0.18, *p* = 0.27	*r* = 0.31, *p* = 0.01^*^
Glucose 120′ OGTT	*r* = 0.25, *p* = 0.12	*r* = 0.62, *p* < 0.01^*^
Insulin 0′ OGTT	*r* = 0.75, *p* < 0.01^*^	*r* = 0.66, *p* < 0.01^*^
Insulin 120′ OGTT	*r* = 0.48, *p* = 0.02^*^	*r* = 0.59, *p* < 0.01^*^
HOMA-IR	*r* = 0.77, *p* < 0.01^*^	*r* = 0.68, *p* < 0.01^*^
Matsuda index	*r* = −0.60, *p* < 0.01^*^	*r* = −0.57, *p* < 0.01^*^

Data are derived from Pearson correlation coefficient. The level of significance was accepted at

* *p < 0.05*.

*VAT, visceral adipose tissue; TG, triglycerides; OGTT, oral glucose tolerance test; HOMA-IR; homeostasis model assessment of insulin resistance*.

In the next step, we created a multiple linear regression model for VAT as a dependent variable. The model that best predicted VAT amount in patients with PCOS included ferritin, insulin at baseline, glucose at the 120 min of OGTT, and TG as independent variables (*p* = 0.02, β = 0.17; *p* = 0.001, β = 0.30; *p* = 0.007, β = 0.26; *p* = 0.001, β = 0.33, respectively). This model explained 69% of the variability in VAT amount (*R*^2^ = 0.687). In the control group, the model that best predicted VAT amount included only HOMA-IR (*p* < 0.001, β = 0.77).

## Discussion

In our study, we demonstrated that higher serum concentration of ferritin is connected with DXA-estimated higher amount of VAT, as well as android and trunk fat mass in PCOS women. This is the first study that shows a relationship between serum levels of ferritin and body composition in the PCOS group estimated by DXA. Additionally, in the regression analysis, the model that best predicted VAT amount in patients with PCOS included ferritin, insulin at baseline, glucose at the 120 min of OGTT, and TG. Interestingly, in the control group, we observed a relationship between serum concentration of ferritin and VAT as well as android fat mass. However, higher serum ferritin concentration and VAT observed in PCOS women in comparison to the control group showed that metabolic disturbance is more profound in this group.

Our finding that ferritin is associated with abdominal obesity corresponds with previous reports in different groups of patients (23). Iwasaki et al. observed a relationship between serum concentration of ferritin and different indices of adiposity measured with computed tomography, e.g., liver fat content, visceral fat area, and subcutaneous fat area in diabetic and non-diabetic subjects. Researchers proposed that serum ferritin level could be a marker of fat distribution, not only visceral fat area, but also subcutaneous fat area (23). In another study, Wu et al. showed an association between serum ferritin concentration and total body fat and trunk fat mass, as well as a negative relationship with leg fat mass (31). The authors performed multivariate regression analysis and found that ferritin levels increased with larger trunk fat mass. In our study, we did not observe an association between serum levels of ferritin and leg fat mass. However, the cited study was conducted in middle-aged and older Chinese (aged 50–70 years), whereas we examined young (mean age 25.1 years) Caucasian women. Interestingly, in a cross-sectional study involving 15,963 Korean males and females, the researchers found increased abdominal obesity across the ferritin concentration quartiles after adjustment for confounders (32). Moreover, there are experimental and human studies demonstrating that ferritin is associated with body fat distribution (23, 31–33). In an experimental study, a close association between overexpression of ferritin and adipocyte differentiation has been shown (33). Accordingly, it has been proposed that ferritin overexpression could represent an adaptive adipocyte response to iron-induced oxidative stress (23). It has been published that in SAT and VAT, increased ferritin protein levels were correlated with SAT and VAT ferritin light polypeptide (*FTL*) gene expression in obese participants (24). Therefore, this could indicate the link between serum ferritin and fat amount in the body shown in PCOS women. However, further studies are needed to clarify the underlying mechanism between serum ferritin concentration and fat distribution in PCOS women.

We showed elevated concentrations of serum ferritin in PCOS women in comparison to the control group. On the contrary, in one study, there were no differences between serum ferritin concentration between the PCOS group and the control group; however, the authors compared groups (PCOS vs. non-PCOS) with different age and BMI (34). In the previous data, increased serum level of ferritin in overweight and obese PCOS women in comparison to the control BMI-matched group has been shown (15, 35). The authors postulated that increased iron body stores, expressed as elevated serum ferritin levels, could be connected with insulin resistance and beta-cell dysfunction (15). On the other hand, the researchers proposed that elevated serum concentration of ferritin could be secondary to the absence of regular blood loss in PCOS women (15); however, in the subsequent study, they did not confirm this hypothesis (35). Accordingly, they found that treatment with metformin, but not anti-androgenic oral contraceptive agents, resulted in a decrease of serum ferritin concentrations and improved insulin sensitivity in PCOS women (35). Apart from insulin resistance and amenorrhea or oligomenorrhea, there could be other potential mechanisms that may participate in iron overload in PCOS women (6). One of them is connected with elevated serum androgens, which could have an impact on erythropoiesis; however, this mechanism has not been confirmed (6). In our study, we observed an association between serum ferritin concentration and FAI. Another hypothesis is that the elevated serum level of ferritin is connected with the presence of oxidative stress, observed in PCOS women (36), which could increase ferritin synthesis to avoid destruction of the cell (37). Moreover, genetic variants related to iron metabolism should be taken under consideration (6). Together, insulin resistance, abdominal obesity, and elevated androgens may also decrease serum hepcidin concentration. It could lead to increased intestinal iron absorption and decreased iron release from macrophages, which leads to overload of iron (6). Additionally, compensatory hyperinsulinemia facilitates iron accumulation within the body (22).

In our study, we also observed an association between serum ferritin and insulin concentration at baseline and at the end of OGTT and with HOMA-IR. Our findings are consistent with previous observation, in which the researchers showed that serum concentration of ferritin is connected with insulin resistance in PCOS women (38). Other studies also reported an association between serum ferritin levels and components of insulin resistance (18, 22). Additionally, an elevated level of serum ferritin, especially in more insulin-resistant subjects, has been shown (6). It is possible that iron overload in the liver may cause insulin resistance by decreasing the inhibiting effect of insulin on hepatic glucose production (22). It has been published that serum ferritin level is positively associated with increased glucose and insulin level (17, 22). Moreover, ferritin as an inflammatory factor is connected with interleukin 6 and tumor necrosis factor alpha, which, in turn, may cause insulin resistance (39, 40). Accordingly, it has been shown that adipose tissue ferritin concentration was inversely associated with insulin receptor substrate 1 gene expression in adipose tissue (24).

The main limitation of the present study is a relatively small sample size, especially regarding the control group.

## Conclusions

On the basis of the obtained results, we concluded that elevated serum ferritin concentration is connected with insulin resistance as well as with DXA-estimated VAT, android, and trunk fat mass in PCOS women and could be a marker of metabolic dysfunction.

## Data Availability Statement

The datasets generated for this study are available on request to the corresponding author.

## Ethics Statement

The studies involving human participants were reviewed and approved by Ethic Committee of Medical University of Białystok, Białystok, Poland. The patients/participants provided their written informed consent to participate in this study.

## Author Contributions

AA: conception and design of the study, acquisition of data, analysis and interpretation of data, and wrote the manuscript. AŁ and AK: acquisition of data. MA: analysis and interpretation of data. IK: analysis and interpretation of data, revising the article, and final approval of the version to be submitted.

### Conflict of Interest

The authors declare that the research was conducted in the absence of any commercial or financial relationships that could be construed as a potential conflict of interest.
